# Dematiaceous fungal endophthalmitis: report of a case and review of the literature

**DOI:** 10.1186/s12348-016-0111-2

**Published:** 2016-11-08

**Authors:** Austin R. Fox, Kourtney H. Houser, William R. Morris, R. Christopher Walton

**Affiliations:** Department of Ophthalmology, University of Tennessee College of Medicine, 930 Madison Ave. Ste. 470, Memphis, TN 38103 USA

**Keywords:** *Pleurostomophora richardsiae*, Dematiaceous fungus, Phaeohyphomycosis, Endogenous endophthalmitis

## Abstract

**Background:**

*Pleurostomophora richardsiae* (formerly *Phialophora richardsiae*) is a dematiaceous fungus that is an uncommon cause of ocular infection. Herein, we present a case of endogenous endophthalmitis associated with disseminated *P. richardsiae* infection.

**Findings:**

This is a descriptive case report with a brief review of literature. A 43-year-old male admitted to the hospital following an acute cerebellar hemorrhage was found to have a swollen and tender wrist. The patient was afebrile with leukocytosis. Visual acuity was hand motion in the right eye and 20/20 in the left. Right eye examination noted anterior chamber cells and flare, vitreous haze and multiple large, and fluffy retinal infiltrates. Diagnostic vitrectomy revealed a mixed inflammatory cell infiltrate with numerous fungal elements. Blood cultures were negative, multiple transesophageal echocardiography studies revealed no vegetations, and synovial fluid aspiration of the wrist and biopsy of the radius were unremarkable. The patient was treated with intravitreal cefazolin, vancomycin, and amphotericin B, topical ciprofloxacin and natamycin, and intravenous amphotericin B and voriconazole. Visual acuity in the right eye declined to light perception, and examination revealed increasing anterior and posterior chamber inflammation. The patient died several weeks after presentation due to a massive intracranial hemorrhage. Fungal culture results from the vitrectomy were received post mortem and were positive for *P. richardsiae*.

**Conclusions:**

*P. richardsiae* endophthalmitis is rare, and outcomes are typically poor. Infections typically occur following traumatic skin inoculation; however, a long refractory period may occur before symptoms develop. Early diagnosis and combination antimicrobial therapy are essential to optimize visual outcomes.

## Findings

### Introduction

Dematiaceous fungi are a heterogeneous group of organisms characterized by the presence of melanin or melanin-like pigment in their cell walls. These pervasive saprobes are commonly found in soil, decomposing plant material, and wood [1, 2]. Phaeohyphomycosis is one of three clinical syndromes caused by these fungi and refers to a spectrum of diseases including superficial and deep local infections, pulmonary infection, and central nervous system infection as well as disseminated disease [1, 2]. Infection typically results following traumatic skin inoculation although many patients do not recall the injury resulting in a long latency period before symptoms develop. Dematiaceous fungi are also an increasing cause of fungal keratitis worldwide [1]. Excluding keratitis, ocular infections with these organisms are uncommon.

The dematiaceous fungus *Pleurostomophora richardsiae* (formerly *Phialophora richardsiae)* has rarely been implicated in human disease. One case of disseminated *P. richardsiae* has been described in the literature in which endophthalmitis was also reported. In this patient, endocarditis was also present as well as positive blood cultures; however, no detailed ophthalmic exam and no vitreous biopsy were described [3]. Herein, we present a case of *P. richardsiae* endogenous endophthalmitis in a patient with disseminated phaeohyphomycosis and briefly review the literature describing endogenous endophthalmitis due to dematiaceous fungi.

## Case report

A 43-year-old man was admitted to the hospital following an acute cerebellar hemorrhage. He complained of recent fever, chills, and left wrist pain. Past medical history included alcoholic cardiomyopathy, porcine mitral valve replacement, chronic atrial fibrillation, and multiple intracranial hemorrhages associated with warfarin toxicity. The chronic left wrist pain and swelling had been previously diagnosed as gout. Social history was notable for heavy ethanol abuse and intravenous drug abuse. Medications upon admission included warfarin, aspirin, metoprolol, and simvastatin. During the preceding four months, he was also treated with systemic corticosteroids following multiple intracerebral hemorrhages.

On admission, the patient was afebrile, and the left wrist was moderately swollen and tender to palpation. His white blood cell count was 16.6 × 10^3^ cells. Blood cultures were negative, and transesophageal echocardiography revealed no vegetations or perivalvular abscess. He was started on intravenous dexamethasone, and 2 days later a craniotomy was performed to evacuate his intracerebral hemorrhage.

Ten days following admission, an arthrocentesis of the left wrist was performed after the patient developed increased swelling and marked worsening of pain. Gram stain revealed numerous white blood cells but no organisms or crystals, and cultures of the synovial fluid were negative. Intrarticular depomedrol was administered following the arthrocentesis.

**Fig. 1:**
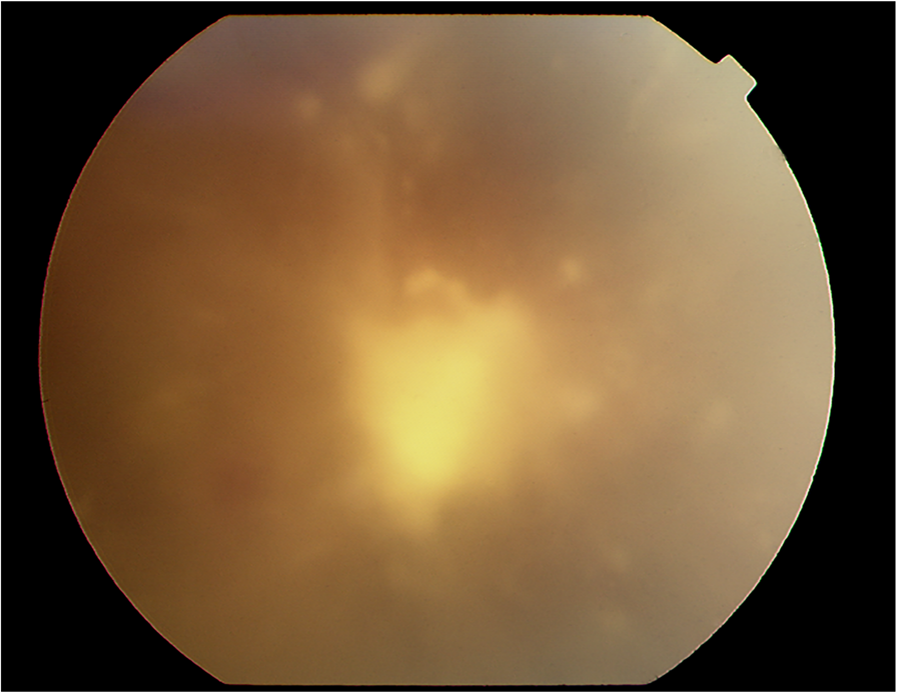
Fundus photograph of right eye showing vitreous inflammation with dense yellow vitreous infiltrate

Two days following the arthrocentesis, the patient complained of right eye pain with redness and blurry vision. Visual acuity was hand movements in the right eye and 20/20 in the left eye. Slit lamp examination revealed severe conjunctival injection, Descemet’s folds, and 3+ anterior chamber cells and flare in the right eye. Moderate vitreous haze with 3+ vitreous cells was noted. Fundus examination revealed multiple large, fluffy retinal and vitreous infiltrates in the right eye (Fig. 1). Examination of the left eye was unremarkable.

**Fig. 2:**
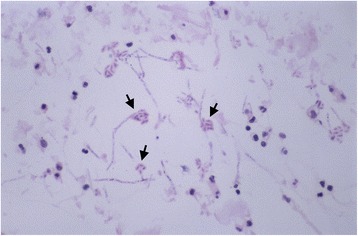
Cytology preparation from vitreous specimen demonstrating numerous white blood cells, hyphal fragments, and clusters of elongated conidia (*arrows*)

A presumptive diagnosis of endogenous endophthalmitis was established. A diagnostic vitrectomy was performed followed by injection of intravitreal cefazolin, vancomycin, and amphotericin B (5 μg). Topical ciprofloxacin, natamycin, and intravenous amphotericin were initiated thereafter. Cytology preparation of the vitreous fluid revealed a mixed inflammatory cell infiltrate with numerous fungal elements including hyphae and clusters of conidia (Fig. 2). Intravenous amphotericin B was continued for the next 10 days; however, the vitritis persisted. During the same period, he complained of worsening left wrist pain and redness. Magnetic resonance imaging of the wrist revealed osteomyelitis of the distal radius, ulna, lunate, and triquetrium. Synovial fluid aspiration and biopsy of the radius was performed, and numerous white blood cells were seen on gram stain, but no bacteria or fungal elements were noted. Transesophageal echocardiography was repeated but again revealed no valvular vegetations.

Subsequently, intravenous voriconazole was added to the treatment regimen but his vision continued to deteriorate to light perception in the right eye. Slit lamp examination showed 4+ cells and flare with a hypopyon, and severe vitritis obscured all fundus details. A repeat vitrectomy was performed, and intravitreal amphotericin B injection (10 μg) was repeated. Microscopic examination of the fluid revealed numerous white blood cells, hyphae, and numerous conidia.

On the following day, the patient died following a massive intracranial hemorrhage. An autopsy was performed, but examination of the eyes was not permitted. Small friable vegetations less than 5 mm in size were present on the insertion ring of the prosthetic mitral valve. Microscopic examination of the vegetations revealed fungal elements including branched septate hyphae and conidia. Multiple organs including the brain, kidneys, and spleen showed evidence of septic embolization. Fungal culture results from the vitreous biopsy were received from the reference laboratory after the patient’s death and were positive for *P. richardsiae*; however, no sensitivity testing was performed.

## Discussion

Over 100 species of dematiaceous fungi have been implicated as the etiology of a spectrum of human diseases [1]. Most of these fungi are considered opportunistic pathogens. Some of the more important genera of dematiaceous fungi include *Alternaria*, *Bipolaris*, *Curvularia*, *Cladophialophora*, *Exophiala*, and *Phaeoacremonium*. Disseminated phaeohyphomycoses is an uncommon disease, typically occurring in immunocompromised or immunosuppressed patients and is associated with a high mortality rate [2, 4].

Endophthalmitis due to dematiaceous fungi is uncommon with a generally poor prognosis [5–13]. Affected eyes typically have devastating visual loss and may ultimately require enucleation. Dematiaceous fungi exhibit slow growth, and therefore, the clinical presentation and diagnosis is often delayed [1]. Many of the dematiaceous fungi are resistant to antifungal therapy; further complicating the management of affected patients. The dematiaceous fungus *P. richardsiae* has rarely been implicated in human disease but has been described in one case of exogenous endophthalmitis following trauma with a retained intraocular foreign body [14].

*P. richardsiae* is a slow-growing dematiaceous fungus that is a common contaminant of decaying wood but can also be isolated from plant material and soil. It forms brown to black colonies with hyaline or brown phialides, which are slender subcylindrical with a flared collarette and spherical, cylindrical, or allantoid conidia at their apex. The phialides grow directly from the branched, septate hyphae or on short branches [15].

Most cases of *P. richardsiae* infections manifest as a subcutaneous granuloma or abscess on the extremities. Infection is thought to result from trauma or inoculation injury such as a splinter, although many cases do not have such a history. This may be due to the long interval between the trauma and development of symptoms and therefore patients may not remember the inciting injury [13, 16]. Rare cases of endocarditis of a porcine mitral valve, dacryocystitis, and exogenous endophthalmitis have also been reported [3, 13, 14]. Similar to other dematiaceous fungi, many cases of *P. richardsiae* infection involve patients who are immunocompromised or immunosuppressed; however, multiple cases have been described in immunocompetent patients [13, 16, 17]. Risk factors for infection include immunosuppression, corticosteroid use, intravenous drug abuse, malignancy, heart valve replacement, and penetrating injury with retained wood material [18].

In our patient, endogenous fungal endophthalmitis was suspected at the time of initial ophthalmic evaluation. Although the source of the disseminated disease in our patient was not apparent, several possibilities seem plausible. The history of chronic wrist swelling and clinical signs of osteomyelitits suggests a subcutaneous inoculation with subsequent prolonged low-grade infection. *P. richardsiae* osteomyelitis has been reported in an HIV-infected patient over 20 years ago; however, such invasive infection is rare [19]. Dissemination from a contaminated prosthetic valve is another possibility since vegetations were ultimately identified on the mitral annulus during the autopsy. A single case of *P. richardsiae* endocarditis in a patient who developed endophthalmitis 4 years after mitral valve replacement has been reported [3]. Although blood and mitral valve cultures grew *P. richardsiae*, aqueous humor culture was sterile, and no mention of vitrectomy was described in this case. Our patient also had multiple risk factors for dissemination including alcohol and intravenous drug abuse and prolonged use of systemic corticosteroids as well as intraarticular injection of corticosteroids.

Most cases of *P. richardsiae* infection are superficial and localized, and surgical excision is typically effective, if not curative [4, 15, 16]. However, antifungal therapy is necessary for more invasive *P. richardsiae* infections, including endophthalmitis [4, 16, 17]. Unfortunately, no universally effective treatment for disseminated disease has been identified, and the mortality rate for such infections is high. Our patient failed to show clinical improvement of his disseminated disease and endophthalmitis despite treatment with systemic and intravitreal as well as topical antifungal agents.

Endogenous endophthalmitis due to dematiaceous fungi is rare and typically is associated with devastating visual outcomes. A literature search using the term endophthalmitis and the currently identified dematiaceous fungi revealed that the most common pathogens were by far *Scedosporium* sp., or the related telomorph *Pseudallescheria boydii*, and *Sporothrix schenckii.* Apart from reports in which these pathogens were implicated, we identified nine other cases of endogenous endophthalmitis due to dematiaceous fungi (Table 1) [5–12]. A history of retained wood, soil, or plant debris following penetrating injury should raise high suspicion for dematiaceous fungal infection, especially in patients with known risk factors. Combination antifungal therapy should be considered as soon as dematiaceous fungi endophthalmitis is suspected due to the potential for devastating visual loss in these patients.

**Table 1:** Reports of dematiaceous fungi in endogenous endophthalmitis^a^

Reference	# cases	Dematiaceous fungi	Related factors	Treatment	Outcome
Rao [5]	1	*Alternaria alternata*	Contaminated intravenous fluids/catheter	Intravitreal: vancomycin, amikacin, amphotericin Intravenous: amphotericin, fluconazole Topical and oral: fluconazole	VA hand movements
Weinberger [6]	1	*Phialemonium curvatum*	Intracavernous injections; diabetes mellitus	Intravitreal amphotericin B	Enucleation
Zayit-Soudry [7]	1	*Phialemonium curvatum*	Intracavernous injections; prosthetic aortic valve; endocarditis	Intravitreal and intravenous: amphotericin B	VA 20/2500
Silva-Vergara [8]	1	*Sporothrix brasiliensis*	HIV infection	Intravenous: amphotericin B Oral: itraconazole	“Bilateral blindness”
Pavan [9]	1	*Bipolaris hawaiiensis*	HIV infection	Intravitreal and intravenous: amphotericin B Oral: fluconazole	Resolved
Wu [10]	1	*Cladosporium* sp.	Postpartum	Intravitreal: fluconazole, amphotericin B, voriconazole	Resolved
Lingappan [11]	1	*Cladophialophora devriesii*	NA	NA	NA
Marangon [12]	2	*Pleurostomophora* sp.	NA	NA	NA
Current report	1	*Pleurostomophora richardsiae*	Mitral valve replacement; systemic steroids; Intravenous drug use	Intravitreal: amphotericin B, cefazolin, and vancomycin Intravenous: amphotericin B and voriconazole Topical: ciprofloxacin and natamycin	VA light perception

Cases include endogenous endophthalmitis due to dematiaceous fungi, not including cases in which the *Scedosporium sp.*, or the related telomorph *Pseudallescheria boydii,* or *Sporothrix schenckii* were implicated

*NA* not available, *HIV* human immunodeficiency virus, *VA* visual acuity

## Abbreviations

HIV: Human immunodeficiency virus
NA: Not available
*P. richardsiae*: *Pleurostomophora richardsiae*
sp*.*: Species
